# Using an Electronic Medication Event–Monitoring System for Antiretroviral Therapy Self-Management Among African American Women Living With HIV in Rural Florida: Cohort Study

**DOI:** 10.2196/14888

**Published:** 2020-02-19

**Authors:** Robert Lucero, Renessa Williams, Tanisia Esalomi, Paula Alexander-Delpech, Christa Cook, Ragnhildur I Bjarnadottir

**Affiliations:** 1 Department of Family, Community, and Health Systems Science Southern HIV & Alcohol Research Consortium University of Florida, College of Nursing Gainesville, FL United States; 2 Department of Family, Community, and Health Systems Science University of Florida, College of Nursing Gainesville, FL United States; 3 College of Nursing University of Central Florida Orlando, FL United States; 4 Department of Family, Community, and Health Systems Science Gainesville, FL United States

**Keywords:** medication adherence, medication therapy management, self-management, HIV, African Americans

## Abstract

**Background:**

HIV remains a significant health issue in the United States and disproportionately affects African Americans. African American women living with HIV (AAWH) experience a particularly high number of barriers when attempting to manage their HIV care, including antiretroviral therapy (ART) adherence. To enable the development and assessment of effective interventions that address these barriers to support ART adherence, there is a critical need to understand more fully the use of objective measures of ART adherence among AAWH, including electronic medication dispensers for real-time surveillance.

**Objective:**

This study aimed to evaluate the use of the Wisepill medication event–monitoring system (MEMS) and compare the objective and subjective measures of ART adherence.

**Methods:**

We conducted a 30-day exploratory pilot study of the MEMS among a convenience sample of community-dwelling AAWH (N=14) in rural Florida. AAWH were trained on the use of the MEMS to determine the feasibility of collecting, capturing, and manipulating the MEMS data for an objective measure of ART adherence. Self-reported sociodemographic information, including a self-reported measure of ART adherence, was also collected from AAWH.

**Results:**

We found that the majority of participants were successful at using the electronic MEMS. Daily use of the MEMS tended to be outside of the usual time participants took their medication. Three 30-day medication event patterns were found that characterized ART adherence, specifically uniform and nonuniform medication adherence and nonuniform medication nonadherence. There were relatively few MEMS disruptions among study participants. Overall, adjusted daily ART adherence was 81.08% and subjective ART adherence was 77.78%.

**Conclusions:**

This pilot study on the use and evaluation of the Wisepill MEMS among AAWH in rural Florida is the first such study in the United States. The findings of this study are encouraging because 10 out of 12 participants consistently used the MEMS, there were relatively few failures, and objective adjusted daily and overall subjective ART adherence were very similar. On the basis of these findings, we think researchers should consider using the Wisepill MEMS in future studies of AAWH and people living with HIV in the United States after taking into account our practical suggestions. The following practical considerations are suggested when measuring objective medication adherence: (1) before using an MEMS, be familiar with the targeted populations’ characteristics; (2) choose an MEMS that aligns with the participants’ day-to-day activities; (3) ensure the MEMS’ features and resulting data support the research goals; (4) assess the match among the user’s ability, wireless features of the MEMS, and the geographic location of the participants; and (5) consider the cost of MEMS and the research budget.

## Introduction

### Background

HIV remains a significant health issue in the United States and disproportionately affects African Americans [1]. African Americans represent 41% of all Americans living with the virus while comprising only 12% of the US population [2,3]. The burden of HIV is highest among African American women with HIV (AAWH) in the southern United States, including Florida, accounting for 63% of all cases in the past 10 years [4]. Among women diagnosed with HIV in 2014, 62% were African American women [3,5]. African American women also account for the largest share of deaths among women with HIV [6]. In 2010, HIV was the leading cause of death for African American women aged 25 to 44 years [7]. Moreover, the HIV mortality rate for African American women in that age group was 10.3 per 100,000 women compared with 0.7 per 100,000 among white women. This was second only to the rate among African American men. For African American women, there appears to be a complex intersection of race, class, and gender, making AAWH one of the most vulnerable groups in the United States [8,9].

The emergence of antiretroviral therapy (ART) has transformed HIV from an acute to a chronic condition, thus allowing individuals to live long lives with HIV [10]. To manage HIV, people living with HIV (PLWH) need to manage medication regimens that demand a high level of adherence. This is in addition to managing symptoms and side effects and various other challenges and barriers to self-management [10]. AAWH experience a particularly high number of barriers when attempting to manage their HIV care [9]. These include community stigma and lack of both general and disease-specific support. In addition, while financial issues, low income, lack of health insurance and other structural barriers in general affect minority populations, these factors disproportionately influence AAWH [9,10]. AAWH often go without care because of limited funds for food, clothing, housing, and other necessities, or postpone care because of lack of transportation [10]. AAWH are also more likely than their white female counterparts to experience unemployment. Those who are employed often report not being able to leave work for medical appointments. When AAWH do access care, a high proportion report not being referred to a case manager and not having enough time with their care provider [10]. Owing to these barriers, AAWH are more likely to rely on the emergency room to receive necessary care and are at higher odds of not receiving prescriptions for ART. In comparison to white women with HIV, a significantly lower proportion of AAWH receive ART or achieve viral suppression [8]. An estimated 50% of AAWH in Florida do not have a suppressed viral load [4]. The reasons for this disparity remain unclear.

To enable the development and assessment of effective interventions that address these barriers to support ART adherence, there is a critical need to understand more fully the use patterns of the objective measures of ART adherence among AAWH. A meta-analysis of the correlations of objective medication adherence via a medication–event monitoring system (MEMS) and self-reported questionnaire revealed that the mean of adherence measured by the MEMS was 74.9% (range 53.4%-92.9%) vs 84.0% by the self-reported questionnaire (range 68.35%-95%) among 11 studies and 1684 PLWH [11]. The correlation between two measures ranged from 0.24 to 0.87. The pooled correlation coefficient for the 11 studies was 0.45 (95% CI 0.34-0.56, *P*=.001), indicating a moderate relationship. There are few studies that report on the actual use patterns that underlie the objective measurement of ART adherence vis-à-vis an MEMS, and none that we know of that compare the objective and subjective ART adherence rates of AAWH [12-14]. Nonetheless, researchers have been steadily increasing the use of MEMS in research among diverse populations [15-22].

### Research Questions

We sought to answer the following research questions in this pilot study:

1. What use patterns of the Wisepill MEMS emerge from the utilization of the system by AAWH?
2. Are there observable differences in an objective measure of ART adherence based on the Wisepill MEMS data and in a subjective measure of ART adherence based on self-reported data among AAWH?

## Methods

### Study Design

As part of a larger mixed method study, we conducted a 30-day pilot study of the Wisepill MEMS among a convenience sample of community-dwelling AAWH in rural Florida. We collected self-reported sociodemographic information and trained AAWH on the use of the MEMS to determine the feasibility of collecting, capturing, and manipulating the MEMS data. In the study reported here, we compared the observational MEMS and self-reported adherence data to address the stated research questions. The qualitative data obtained, not reported here, are being analyzed separately to address a research question related to MEMS use and HIV-related stigma.

### Medication Event Monitoring via the Wisepill Dispenser

**Figure 1:**
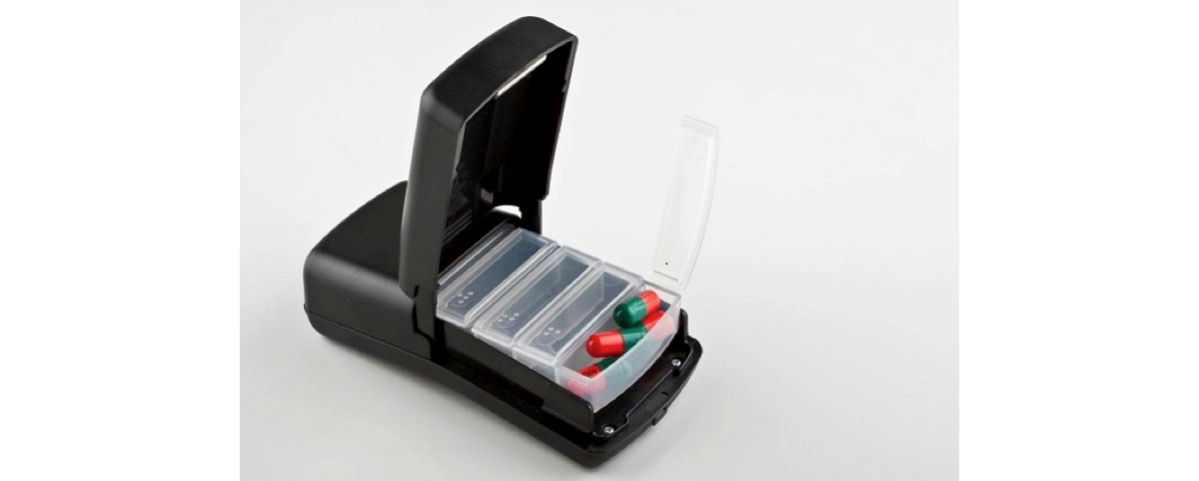
Wisepill RT2000 medication event–monitoring system.

The Wisepill MEMS was chosen from among other MEMS based on the ability to organize daily medication events, as shown in Figure 1, and system design (ie, not a pill bottle) in an attempt to avoid inducing stigma. In this pilot study, we trained AAWH to use the Wisepill MEMS. A 1100 mA lithium polymer rechargeable battery provides power to the Wisepill RT2000, which holds approximately 30 large pills or 60 small pills in a seven-compartment inner container. Each time the compartment is opened, a cellular signal is sent and recorded in real-time on a Web-based server. Each Wisepill device contains a subscriber identity module, and the transmission of data is primarily by general packet radio service to the server. Data transfer may also occur via SMS. However, general packet radio service is preferred to short message service because (1) it is less expensive and (2) the server deletes the data after it receives it. In addition to recording device openings, the Wisepill signal reports the remaining battery power for the device, airtime on the subscriber identity module, and strength of the signal. In a signaling subsystem, nonvolatile, electrical, erasable, programmable, read-only memory maintains data for later transmission if there is a power failure and connectivity is lost. The data are immediately accessible to research staff via a secure internet interface.

**Figure 2:**
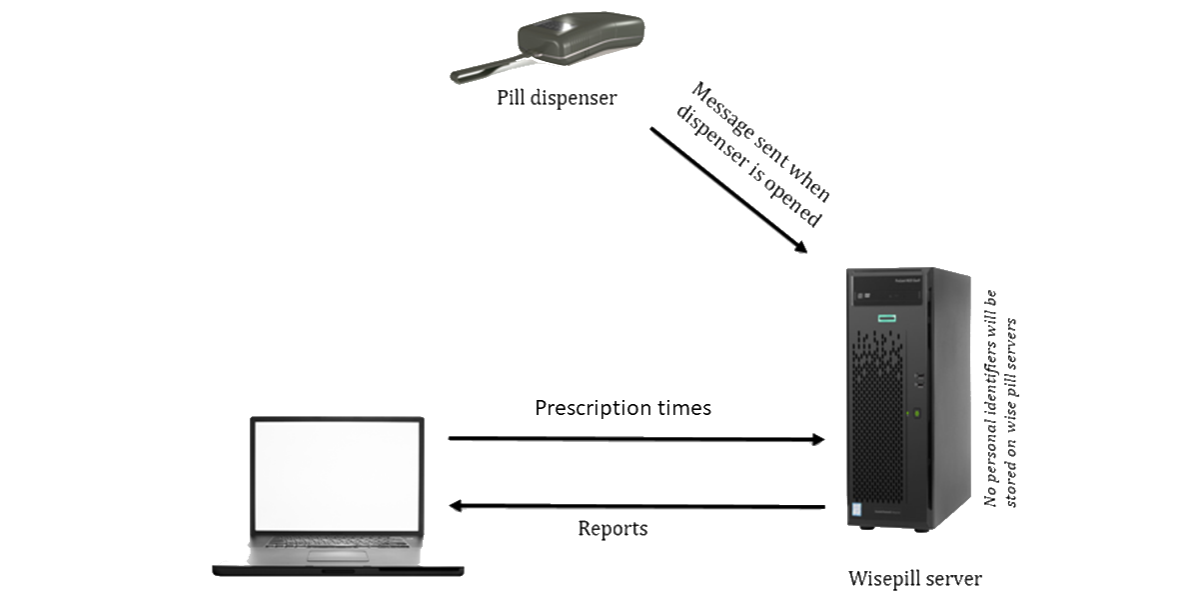
Wisepill medication event–monitoring system data capture and collection.

Figure 2 depicts the flow of information captured and stored on the Wisepill secured server as well as information flow between the Wisepill client, or research site, and the Wisepill secured server. The research site programs each participant’s prescription times using a unique identifier for each Wisepill user. In addition to automated downloadable reports from the Wisepill server, discreet medication events can be imported from the Wisepill server via a CSV file for individual-level analyses.

### Recruitment

We recruited community-dwelling AAWH using two approaches: (1) searched two research registries (ie, the Florida Cohort Study and HealthStreet) that contain information on PLWH who may have consented to share their information for research opportunities and (2) collaborated with support groups that serve PLWH. The Florida Cohort Study began in 2014 and collects demographic, behavioral, and social factors affecting health outcomes for PLWH within the state of Florida. Any person with HIV aged 18 years or older is eligible to participate in the study. As part of the informed consent process, individuals can agree to participate in future research studies. In addition, HealthStreet is a community engagement program at the University of Florida that aims to improve the health of community members by bridging health care and health research. Community health workers assess health concerns, conditions, and research perceptions of community members, and provide referrals to community members for medical and social services, as well as opportunities to participate in health research. Finally, we attended HIV support group meetings regularly, presented our proposed study, and recruited interested individuals. The University of Florida institutional review board approved this study.

AAWH were eligible to participate in the study if they met the following criteria: (1) age between 18 and 55 years, (2) female, (3) on ART, (4) willing to use an electronic monitoring event system to monitor adherence to one of their ARTs, and (5) not an employee or a student of the University of Florida. Participants received up to US $75 on a cash card for their time and effort upon completion of the 30-day study period.

### Data Collection

Once a member of the research team determined a potential participant’s eligibility, we met with the participant during an agreed upon date and time to obtain consent, conduct training on the use of the Wisepill MEMS, and collect baseline measures including sociodemographic characteristics. After completing the baseline measures and MEMS use training, the research team member provided the participant US $25 cash card for time and travel expenses. After 15 days of using the MEMS, a research team member followed up with participants by telephone to ask how the participant was doing with the use of the MEMS. We added US $25 to a participant’s cash card when a member of the research team successfully contacted them by telephone. The telephone call was intended to troubleshoot potential technical barriers, and no additional data were collected from participants. At the end of the 30-day study period, a member of the research team met with each participant to collect the follow-up self-reported data. After the meeting was completed, we added a final US $25 to the participant’s cash card.

### Data Analysis

Baseline and follow-up self-reported data were stored in a secured Research Electronic Data Capture database at the University of Florida and exported to a Microsoft Excel file for analysis. The MEMS data are stored in the Wisepill secured server. Self-reported data were evaluated and reported using descriptive statistics appropriate for measurement level (eg, mean, median, standard deviations, frequencies, percent, and range). We checked for implausible or out-of-range values, distributional forms, and missingness.

#### Wisepill Use Patterns Among African American Women With HIV

We evaluated the participant’s Wisepill MEMS data transmission to identify scheduled, unscheduled, and missed medication events as well as MEMS disruptions. *Disruptions* could occur because of battery failure, forwarder malfunction, or a participant’s decision not to use the MEMS. *Scheduled medication events* were captured when a participant opened the MEMS during a 1-hour period that was typical of their daily dosing pattern. We used the 1-hour period based on the *7 rights* of safe medication preparation and administration, including right time [23]. A research team member programmed the scheduled medication event or events within the Wisepill research interface according to the participant’s self-reported information. An *unscheduled medication event* occurred when a participant opened the MEMS outside of a scheduled medication event period. *Extra medication events* were logged when a participant opened the MEMS more than once during a scheduled medication event period. A *missed medication event* was recorded when participants neglected to open the MEMS during a scheduled medication event period. Using summary statistics (ie, range, mean, and median), we characterized the data transmission patterns of medication events (ie, scheduled, unscheduled, and extra), missed medication events, and disruptions for the entire sample.

We generated graphical dot plots of each participant’s 30-day medication events, including scheduled, unscheduled, and extra medication events. After printing each dot plot, two members of the research team (RL and RW) reviewed the collection of dot plots on an open table to identify and create categories of patterns that could emerge from the 30-day medication events among study participants. We utilized a consensus process that included one other research team member (PD) to settle disagreement between RL and RW about a 30-day medication event pattern.

#### Objective and Subjective Antiretroviral Therapy Adherence Among African American Women With HIV

##### Objective Antiretroviral Therapy Adherence

Our primary outcome was an objective measure of ART adherence using the Wisepill MEMS. We measured objective ART adherence using two approaches: (1) scheduled ART adherence or the proportion of scheduled medication events during the study period (ie, total number of scheduled medication events/30 dosing events), and (2) daily ART adherence or the proportion of scheduled and unscheduled medication events during the study period (ie, total number of scheduled + unscheduled medication events/30 dosing events). In addition, given advances in wireless connectivity, it was unlikely that there would be any system failures. However, we also considered a separate calculation of objective ART adherence that would account for disruptions (ie, loss of wireless signal or technical failure) in receiving data. We censored technical failures and considered the absence of a clear technical failure a missed medication event [24].

##### Subjective Antiretroviral Therapy Adherence

Our secondary outcome measure was subjective ART adherence using a validated three-item self-report instrument that assesses adherence during the previous 30 days (Cronbach alpha: .86-.89) [25]. The three items include (1) How many days did you miss at least one dose of any of your HIV medicines? (response options: 0-30), (2) How good a job did you do at taking your HIV medicines in the way you were supposed to? (response options: very poor/poor/fair/good/very good/excellent), and (3) How often did you take your HIV medicines in the way you were supposed to? (response options: never/rarely/sometimes/usually/almost always/always). We calculated a proportion of the total number of days participants successfully took their medication based on the answer to question 1 (eg, two missed doses: 28/30=98%).We assigned adherence in 20% increments to the response categories in questions 2 and 3 (eg, very poor=0%, poor=20%, and fair=40%) [26]. We report the mean and median of overall adherence to each question and an overall summary of ART adherence by taking an average of the three questions answered and an aggregate across participants.

## Results

### Participant’s Characteristics

As the eligibility criteria were applied directly to the existing consent-to-share registries (ie, the Florida Cohort Study and HealthStreet) and the criteria were shared at support group meetings, no one was excluded from the study because they were not eligible. We had a refusal rate of 76% across the three recruitment strategies. A total of 14 AAWH participated in this pilot study. All participants, except for one, completed the 30-day follow-up meeting with a member of the research team. Table 1 contains summary statistics of the study participants’ characteristics who completed the study. The average age of the study participants was 49 years with a range from 23 to 63 years. Nearly 46% (6/13) of the participants were not a high school graduate or did not have a general educational diploma. Only 3 of the participants were married or living with a long-term partner. As we were interested in personal technology use among our study participants, a set of internet use questions were included among baseline measures. It appears that at least 10 participants owned and used their own computer, laptop, or tablet, and 7 participants owned and used a mobile phone for internet activity. Notably, at least eight participants relied on public access computing to access the internet.

**Table 1:** Summary of study participants’ characteristics (N=13).

Characteristics				Values
Age (years), mean (SD), range				48.9 (11.5), 23-63
**Highest grade/year of school completed, n (%)**				
	Some high school (grades 9-12)		5 (39)	
	High school graduate or general education diploma		5 (39)	
	Some college or technical/trade school		1 (8)	
	College or trade school graduate		2 (15)	
**Marital status, n (%)**				
	Married		3 (23)	
	Divorced		2 (15)	
	Widowed		1 (8)	
	Separated		2 (15)	
	Never married/single		5 (39)	
	Living with a long-term partner		0 (0)	
**Primary use of the internet, n (%)**				
	**Own computer/laptop/tablet at home**			
		Never		3 (23)
		Rarely		1 (8)
		About once a week		1 (8)
		A few times a week		4 (31)
		Daily		4 (31)
	**Computer at public locations (eg, library)**			
		Never		5 (39)
		Rarely		5 (39)
		About once a week		1 (8)
		A few times a week		2 (15)
		Daily		0 (0)
	**Mobile phone**			
		Never		5 (39)
		Rarely		5 (39)
		About once a week		1 (8)
		A few times a week		2 (15)
		Daily		0 (0)

### Medication Event Patterns

There was 510 expected medication event days for the study sample, which includes multiple scheduled medication events per day for 3 participants. We observed 144 scheduled, 216 unscheduled, one extra, and 366 missed medication events among the 14 participants, not including disruptions. We also detected disruptions with two of the participant’s use of the Wisepill MEMS. These disruptions were because of battery failures, which resulted in 41 disruption days.

**Figure 3:**
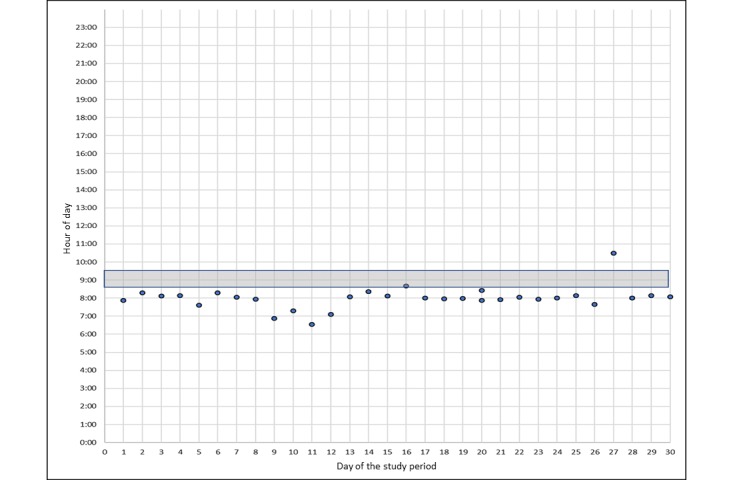
Uniform medication adherence.

**Figure 4:**
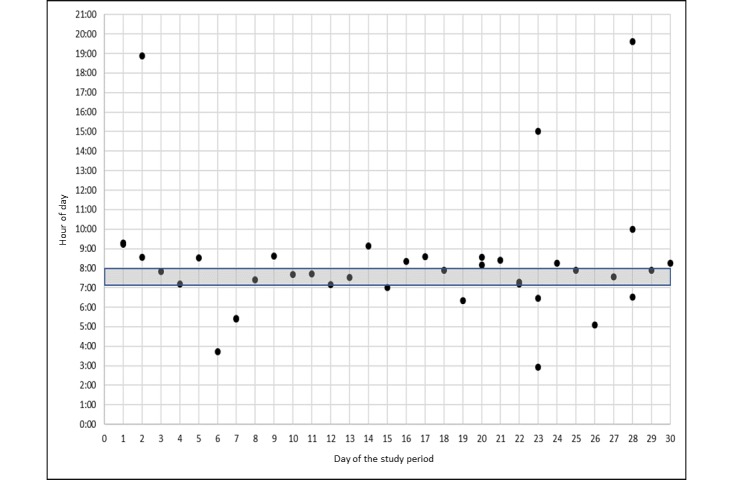
Nonuniform medication adherence.

**Figure 5:**
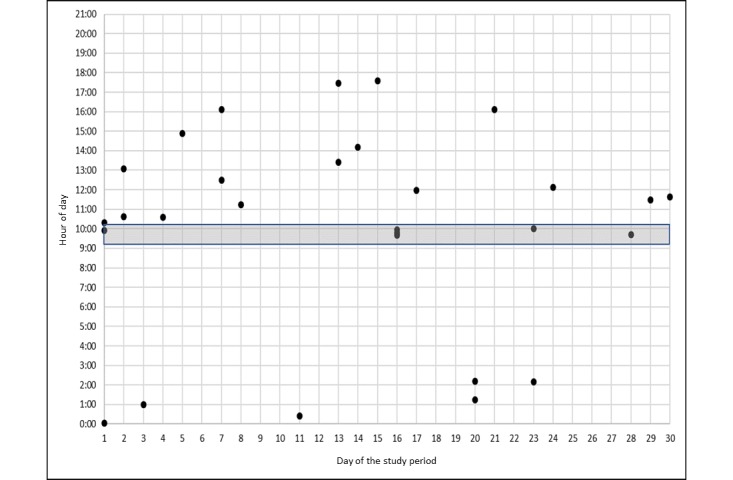
Nonuniform medication nonadherence.

We identified three patterns of 30-day medication events among the graphical dot plots of 12 participants and 14 scheduled medication events. The 2 participants who had a disrupted experience with the Wisepill MEMS were not included in this analysis. Figures 3-5 represent uniform and nonuniform medication adherence and nonuniform medication nonadherence, respectively. The grey horizontal bar indicates the time in which the participant reported usually taking their daily ART. Each dot is a scheduled, unscheduled, or extra medication event. In all, 5 participants had uniform medication adherence or took their ART mostly (ie, ≥24 events) during a uniform range of time (see Figure 3). Of these 5 participants, 1 had two uniform medication adherence patterns. A total of 5 participants had nonuniform medication adherence or mostly took (ie, ≥24 events) their ART but not in a uniform range of time (see Figure 4). Finally, 3 participants had nonuniform medication nonadherence or did not take their ART during a uniform range of time and missed a substantial number of ART events (ie, ≥10 events; see Figure 5). None of the participants had uniform medication adherence that was consistent with the time they reported usually taking their daily ART.

### Antiretroviral Therapy Adherence

Table 2 contains a summary of the objective and subjective ART adherence measures we constructed from the observations captured using the Wisepill MEMS and the self-reported data of AAWH. There was a wide variation in the use of the MEMS, and extreme differences in the scheduled and daily ART adherence. On average, study participants used the MEMS with around 28% (3/10) of scheduled medication events during the study period. Some participants either chose not to or were unable to adhere to their usual medication schedule while others were able to with 67% (2/3) of events. However, based on adjusted scheduled and unscheduled daily medication events, participants on average used the MEMS at 81% (8/10) of events and some up to 100% for 30 days.

In comparison, the average number of days that participants self-reported not taking their ART was 2.05, or on average 93.17% adherence during the 30 days. Participants reported that they generally did a good job taking their ART 70.8% of the time. On the other hand, participants reported that sometimes (ie, 64.62%) they consistently took their ART as prescribed. On the basis of these self-reported behaviors, participants in general were 77.78% adherent to the ART.

**Table 2:** Objective and subjective antiretroviral therapy adherence among a pilot sample of African American women with HIV.

Adherence measures					Values
**Objective measures of adherence using the Wisepill MEMS^a^ for 30 days (N=12)**					
	**Scheduled ART^b^ adherence, mean (SD), range**				
		Unadjusted		28.24 (33.33), 0-66.67	
		Adjusted		28.24 (33.33), 0-66.67	
	**Daily ART adherence, mean (SD), range**				
		Unadjusted		70.59 (83.33), 10.00-100.00	
		Adjusted		81.08 (86.67), 26.67-100.00	
**Three-item self-report measure of adherence^c^(N=13)**					
	Overall subjective ART adherence, mean (SD), range				77.78 (80.00), 56.67-100.00
	**Individual-item subjective ART adherence**				
		**1. In the last 30 days, on how many days did you miss at least one dose of any of your antiretroviral medication?^d^, n**			
			Did not miss	9	
			1 day	2	
			2 days	0	
			3 days	2	
		Overall adherence for item 1 (0-100), mean (median)^e^		97.95 (100)	
		**2. In the last 30 days, how good a job did you do at taking your antiretroviral medication in the way that you were supposed to?, n**			
			Very poor	0	
			Poor	0	
			Fair	4	
			Good	2	
			Very good	3	
			Excellent	4	
		Overall adherence for item 2 (0-100), mean (median)^e^		70.77 (80)	
		**3. In the last 30 days, how often did you take your antiretroviral medication in the way that you were supposed to?, n**			
			Never	1	
			Rarely	1	
			Sometimes	1	
			Usually	3	
			Almost always	5	
			Always	2	
		Overall adherence for item 3 (0-100), mean (median)^e^		64.62 (80)	

^a^MEMS: medication event–monitoring system.

^b^ART: antiretroviral therapy.

^c^The total number of responses for each question are based on all participants except for one who did not complete the end-of-study data collection meeting.

^d^The range of responses for this question is based on the minimum and maximum missed doses reported by participants.

^e^We calculated a proportion of the total number of days participants successfully took their medication in question 1 (eg, two missed doses: 28/30=98.3%). We assigned adherence in 20% increments to the response categories in questions 2 and 3 (eg, very poor=0%, poor=20%, and fair=40%).

## Discussion

### Principal Findings

We found among a sample of AAWH in rural Florida who were mostly high school graduates, unmarried, and owned some consumer technology that a majority were successful at using an electronic MEMS. Even though participants had a typical time to take their ART, their daily use of the MEMS tended to be outside of this usual time. None of the participants used the MEMS 100% of the time for scheduled medication events compared with 4 participants who successfully used the device 100% for daily medication events. This variation in medication administration was also identified among the studies including in the meta-analysis of ART adherence by Shi et al [11]. The barriers that have been reported to influence ART adherence may also modify the ability of AAWH to maintain a typical time of day to take their medication.

We identified three 30-day medication event patterns that characterized ART adherence among participants, namely, uniform and nonuniform medication adherence and nonuniform medication nonadherence. This finding could be important for the development of targeted self-management interventions based on the three use patterns [27-29]. In other words, the identification of an underlying medication event pattern can inform goal setting, action planning, or problem solving based on some observed reality.

Although few participants self-reported less than 100% ART adherence, a much smaller proportion thought they did a good job repeatedly taking ART as prescribed. These findings are nearly the same as reported by Shi et al [11]. On average, the self-reported ART adherence in this study was similar to the adjusted objective ART adherence. Unlike other studies, on average, our participants had greater objective ART adherence than self-reported ART adherence. This may be, in part, because of the difference in creating a quantitative measure from categorical data.

### Limitations

This study provides important foundational insights into the use of an electronic MEMS to determine an objective measure of ART adherence among African American women who are disproportionately affected by HIV. However, there were limitations to our pilot study, and we acknowledge the small sample size. This was the first study conducted by the authors using the Wisepill MEMS. This may have unduly led to disruptions or technical failures that might not have otherwise occurred with experienced investigators or users. Second, we recruited a convenience sample of AAWH to use the MEMS. The participants may have been motivated to use the MEMS because of social desirability, which can occur when an individual’s behavior is favorable to others. However, given the variation in the scheduled and daily ART adherence and self-reported ART adherence summary measures, there appears to be minimal influence on the participant’s behavior by intrinsic (eg, desire to appear more adherent to ART than usual) or extrinsic (ie, baseline and 15- and 30-day US $25 compensation) motivators.

### Comparison With Previous Work

Previous studies that have focused on the feasibility of using the Wisepill MEMS to monitor ART adherence occurred mostly in settings outside of the United States [14]. In Uganda, researchers found that the Wisepill MEMS produced similar results as that of MEMS pill bottle cap, and male (n=2) and female (n=8) study participants described the device as easy to use and convenient [24]. Another group of researchers in China found that, although using the Wisepill MEMS for real-time medication monitoring was technically feasible with men (n=2) and women (n=8), there were concerns regarding the acceptability of the device to patients [13]. Only half of all participants reported positive experiences. Researchers in Tanzania showed that real-time medication monitoring using the Wisepill MEMS was both a feasible and acceptable way to measure ART adherence among men (n=2) and women (n=3) [12].

To the best of our knowledge, ours is the first study to report empirical data on system use of the Wisepill MEMS in the United States even though the device is being used increasingly by researchers to construct an objective measure of ART adherence [15-22]. Similar to studies conducted in other countries, our experience was not a perfect one. Not all of our participants appeared to find the Wisepill MEMS easy to use. This may have been partly because of the battery failures we identified and also because none of our participants used the MEMS 100% of the times during scheduled events. Therefore, pretesting devices is necessary before releasing the MEMS to study participants. The fact that the majority of our participants were not able to use the MEMS uniformly could suggest something about the overall usability of the device’s design.

A recent laboratory study assessed the accuracy of 10 commercially available MEMS, including Wisepill [30]. The researchers measured the accuracy of three devices and defined this parameter as scheduled events that fell within 120 seconds of the date and time recorded on paper for three devices. Of the 10 MEMS, 7 accurately registered ≥96% of the scheduled events across the three devices while the Wisepill device did so with accuracy at 100%, 92%, and 84%. We concur with McGrady’s [30] conclusion that the *best* MEMS depends on the research study and population sample. We also suggest the following practical considerations when measuring objective medication adherence: (1) before using the MEMS, be familiar with the targeted populations’ characteristics; (2) choose an MEMS that aligns with the participants’ day-to-day activities; (3) ensure the MEMS’ features and resulting data support the research goals; (4) assess the match among the user’s ability, wireless features of the MEMS, and the geographic location of the participants; and (5) consider the cost of MEMS and the research budget. Although we did not necessarily follow these simple considerations before using the Wisepill MEMS, our study results are encouraging because 10 out of 12 participants had consistent use of the MEMS, there were relatively few disruptions in the device use, and objective adjusted daily and overall subjective ART adherence were very similar in our study.

### Conclusions

This pilot study on the use and evaluation of the Wisepill MEMS among AAWH in rural Florida is the first such study in the United States. We found that AAWH were generally successful at using the MEMS for 30 days. Overall adjusted daily ART adherence was 81.08% and subjective ART adherence was 77.78%. The use of the MEMS among study participants resulted in three clear patterns of behavior: uniform and nonuniform medication adherence and nonuniform medication nonadherence. In summary, we think researchers should consider using the Wisepill MEMS in future studies of AAWH and PLWH in the United States after considering our practical suggestions.

## Abbreviations

AAWH: African American women with HIV
ART: antiretroviral therapy
MEMS: medication event–monitoring system
NIH: National Institutes of Health
PLWH: people living with HIV
